# Effect of fruit and mint flavored Rogue^®^ oral nicotine product use on smoking reduction and quitting in a 6-Month prospective cohort of adults who smoke cigarettes

**DOI:** 10.1186/s12889-024-20463-3

**Published:** 2024-11-22

**Authors:** Elliott H. McDowell, Jason N. Kennedy, Michael Feehan, Stacey A. Bell, Sarah E. Marking, Jessica P. Zdinak, Andrew R. Joyce, Michelle Humphreys

**Affiliations:** 1 Oracle Life Sciences, Oracle Corporation, 2300 Oracle Way, Austin, TX 78741 USA; 2 M/A/R/C Research, 1425 Greenway Drive, Suite 300, Irving, TX 75038 USA; 3 Department of Ophthalmology, Jacobs School of Medicine and Biomedical Sciences, Ross Eye Institute, State University of New York at Buffalo, 955 Main Street, Buffalo, NY 14203 USA; 4 Sanova, 1806 Summit Ave Suite 300 #288, Richmond, VA 23230 USA; 5 Applied Research and Analysis Company, 3208 Nutley Court, Henrico, VA 23233 USA

**Keywords:** Oral nicotine products, Nicotine pouches, Flavored tobacco products, Smoking reduction and quitting, Switching behaviors

## Abstract

**Background:**

Quitting cigarette smoking can substantially reduce or eliminate the risk of developing numerous chronic diseases. Use of flavored tobacco or nicotine products is commonly cited by adults who smoke cigarettes to be important in helping them reduce or quit smoking. The purpose of this analysis was to understand the association between the levels of use of flavored oral nicotine products and smoking reduction and quitting and how reduction or quitting may differ between predominant users of fruit/other versus mint flavored oral nicotine products after six months of use.

**Methods:**

Participants were provided with their choice of a variety of forms and flavors of Rogue^®^ nicotine products (Study Products) over a 6-month actual use period and completed online surveys assessing tobacco, nicotine and Study Product use at Baseline and Months 1, 2, 4, and 6 thereafter.

**Results:**

Among the 1393 participants at Month 6, 41.4% and 52.5% used predominantly fruit/other or mint Study Product flavors, respectively. Compared to predominant mint users, predominant fruit/other users had greater cigarette reduction (mean reduction: 50.0% vs. 48.4%) and a higher proportion had quit smoking (proportion quit: 15.4% vs. 11.6%) at Month 6. Additionally, 38.8% of predominant fruit/other users and 39.3% of predominant mint users reduced their cigarette consumption by *≥* 50% from Baseline. Increased use of fruit/other flavors was independently associated with smoking reduction (8.6% greater reduction per 10 pieces/day; *p* < 0.001) and odds of quitting smoking (OR = 1.29 [95% CI: 1.04–1.59] per 10 pieces/day; *p* = 0.017). Increased use of mint flavors was independently associated with smoking reduction (7.5% greater reduction per 10 mint pieces/day; *p* < 0.001) but not with odds of quitting smoking.

**Conclusions:**

Increased use of either fruit/other or mint flavored Study Products at Month 6 was associated with significantly increased smoking reduction, whereas only increased use of fruit/other flavors was associated with greater odds of quitting smoking among participants in the study.

**Trial Registration:**

This study was observational. Participants were not prospectively assigned to one or more health-related interventions and could choose to use or not use the commercially available study products provided during the study. Thus, the study was not registered in a trial database by the Sponsor.

**Supplementary Information:**

The online version contains supplementary material available at 10.1186/s12889-024-20463-3.

## Introduction

Quitting cigarette smoking can substantially reduce or eliminate the risk of developing numerous chronic diseases [1–4] among the estimated 28.3 million adults who smoke cigarettes (AS) in the United States (U.S.) [5]. Changing smoking behavior can be difficult, and some AS are unable to quit using conventional methods [6–9]. Instead, AS are increasingly adopting novel, reduced-harm tobacco and nicotine products (TNP) to reduce or replace smoking cigarettes [10–15].

Current and former AS report that they use these novel TNP, in part, due to the availability of nontobacco, nonmenthol flavors (hereafter, flavors/flavored) and consider flavors to be important in helping them transition from cigarettes [16, 17]. One category of novel TNP that AS report using for this purpose are oral nicotine products (ONP), which are commercially available in a variety of flavors in the U.S. market [16, 18–21]. Though substantial evidence exists for the effect of flavor use on smoking reduction and quitting for other novel TNP (e.g., electronic nicotine delivery systems [ENDS]) [22–30], comparable evidence specific to ONP use is sparse.

The research that is available on ONP and switching behaviors are based on cross-sectional surveys [31], secondary analyses of results from small-sample pharmacological studies [32], and short-term (4- or 6-week), site-based actual use studies [33–36]. Results from one study on *ad libitum* use of nicotine pouches found that using more flavor varieties over 6 weeks was associated with increased cigarette reduction [36]. Otherwise, no published, longer term actual use studies to date have reported on the association between flavored ONP use and changes in cigarette smoking. Ultimately, there is a need for high-quality, longitudinal switching studies or controlled trials to understand the effect of flavored ONP.

The aim of the study was to understand how ONP use was associated with smoking reduction and quitting after six months. Overall results demonstrated that ONP use was significantly associated with smoking reduction and quitting (mean reduction in cigarettes per day (CPD): 49.7%; proportion quit: 13.3%; *p* < 0.001 for both).

The purpose of the present analysis was to understand the degree of association between the level of use (variety and amount) of flavored ONP (fruit/other and mint flavors) and smoking reduction and quitting over six months. In addition, this analysis was also designed to understand differences in reduction or quitting smoking between predominant and exclusive users of fruit/other or mint flavored ONP, which represent nontraditional and traditional flavors, respectively, within the greater oral/smokeless tobacco category. Results of this analysis can help address current gaps in knowledge regarding the effect of flavored ONP use on transitioning from cigarette smoking.

## Methods

### Study design

The study was a 6-month prospective cohort study of age verified AS (ages *≥* 21 years). Candidate participants were recruited from a market research panel of adults who used TNP (Qualtrics; Provo, UT) and were screened for eligibility prior to enrollment. All participants agreed to an electronic informed consent form and were compensated for their participation. The first participant was enrolled on 01 April 2022 and the last participant completed the study on 26 October 2022.

The study was conducted in accordance with the Declaration of Helsinki. The study and all materials were approved by the Sterling Institutional Review Board (Atlanta, GA) prior to data collection (Study ID 9741; Approved 28 February 2022). Oracle Life Sciences (Oracle Corporation; Austin, TX; formerly, Cerner Enviza) had oversight of all study procedures.

### Participant recruitment and enrollment

The size of the target cohort at baseline was 2000 adult (ages *≥* 21 years) participants. Prior research suggested this was sufficient to characterize potential changes in cigarette smoking. Candidate participants were screened against pre-defined inclusion and exclusion criteria.

Participants were required to: be *≥* 21 years old; currently smoke cigarettes (smoked 100 + cigarettes in their lifetime and smoked every day or some days in the past 30 days [P30D]); consent to have their age verified by a third-party vendor (Veratad Technologies; Teaneck, NJ); be able to read and understand English; reside in the contiguous U.S.; consider themselves in generally good health; be open to trying ONP; have access to the internet via a computer or mobile device; provide informed consent; and agree to study requirements. Participants could not: be residents of Arkansas, Massachusetts, Utah, Maine, or Vermont (due to TNP shipping restrictions); be currently pregnant or breastfeeding; be employed by a TNP company or living with or related to someone who is employed by a TNP company; have taken a survey about TNP use in the past two weeks. Recruitment and enrollment took four weeks and invitations to follow-up surveys were sent to participants based on the date they enrolled.

### Study products

Enrolled participants were provided with their choice of a variety of Rogue^®^ nicotine products, hereafter referenced as Study Products (SP), during a 6-month actual use period (AUP). Select products from the SP’s commercially available portfolio were made available to participants in three forms: Pouch (6 mg nicotine), Gum (4 mg), and Lozenge (4 mg). SP were available during the study in ten varieties of fruit/other and mint flavors across three forms. Products were provided to participants at no cost. Specific flavors available for each form are listed in Table 1.

**Table 1:**
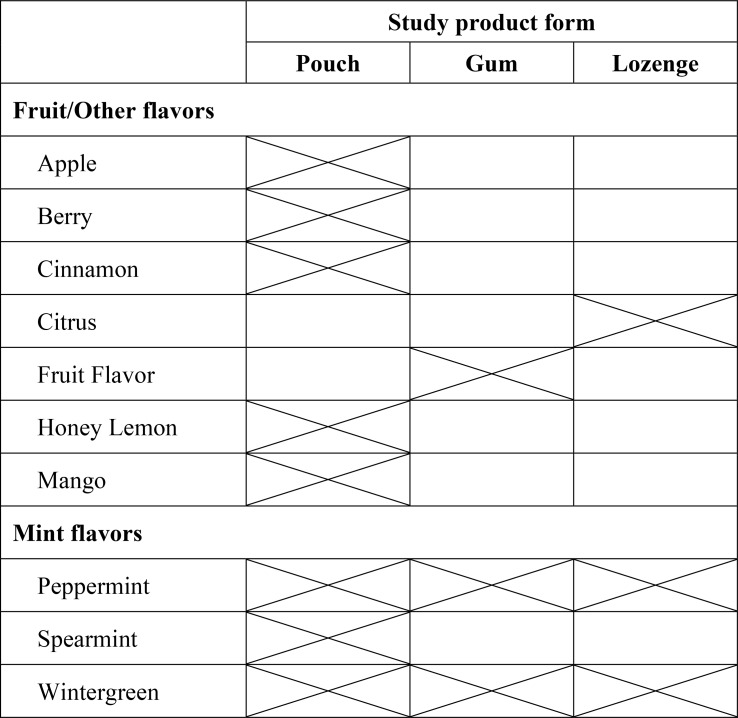
Flavor varieties of Study products (SP) made available to participants

### Study procedures

Online surveys (approximately 15 min in duration) assessing TNP and SP use were conducted at enrollment (Baseline) and Month 1, 2, 4, and 6. Participants used an online product order form to select the SP they wished to use during each month of the AUP. Product orders were fulfilled and shipped by Rogue Holdings, LLC (the Sponsor) at no cost to participants. Participants were required to provide proof of age (*≥* 21 years old) at the time of delivery. Participants were instructed to dispose of unused SP at the end of the AUP.

Participants could withdraw from the study or could be discontinued by the investigators if they missed two consecutive surveys, could not have their SP delivered, or if there was a risk to their safety (e.g., reported an adverse event to the Sponsor).

### Measures and definitions

Data collected during the study included participant demographic characteristics and past-30-day (P30D) cigarette, SP, and other TNP use. Survey items were adapted from the PATH study and other federal surveys on TNP use [37–39].

#### Measures

Participants reported P30D frequency of use (‘In the past 30 days, did you smoke/use other TNP/use Rogue every day, some days, or not at all?’), the number of days used (‘On how many days in the past 30 days did you smoke cigarettes/use Rogue [flavor-form combination]?’), and the quantity used per day for cigarettes and SP (‘How many cigarettes/pieces of Rogue [flavor-form combination] did you usually smoke/use each day?’) during each survey. Participants were only asked to report frequency of use for any other TNP they used in the P30D. Definitions and examples of the other TNP assessed (ENDS, smokeless tobacco, ONP, nicotine replacement therapies (NRT), and non-cigarette smokeable tobacco products) were based on those used in the PATH study [37]. Usual use of menthol or nonmenthol cigarettes was assessed at Baseline (‘During the past 30 days were the cigarettes that you usually smoked menthol?’).

#### Study product flavor use and smoking outcome definitions

The number of SP flavors used by a participant was defined as the number of distinct flavors a subject reported using in the P30D, regardless of form. The number of form-flavor combinations used by a participant was defined as the number of distinct combinations of form and flavor used by a participant.

Predominant use of SP flavors was defined by the relative quantity (i.e., number of pieces) of fruit/other or mint flavored SP used in the P30D, with participants assigned to the category with greatest use. Equivalent use was defined as using the same quantity of fruit/other or mint flavored SP. Exclusive use was defined as using only fruit/other or mint SP flavors. These definitions are based only on SP flavors used, irrespective of SP form.

Percent reduction in cigarette smoking was defined as the percent change in the total quantity of cigarettes smoked in the P30D at Month 6 versus Baseline. Quitting smoking was defined as reporting having smoked ‘not at all’ in the P30D at Month 6.

### Data analysis

Outcomes, exposures, and subgroup analyses were specified a priori for all analyses.

#### Association between Variety of SP flavors used and smoking outcomes

Bivariate linear regression was used to quantify the association between patterns of SP flavor use and percent reduction in quantity of cigarettes smoked in the P30D. Bivariate logistic regression was used to quantify the degree of association between each exposure variable and the likelihood of quitting vs. not quitting smoking. Exposure variables were: (1) the number of unique SP flavors used in the P30D, including the number of individual flavors (up to 10 flavors) and (2) the number of unique SP form-flavor combinations used (up to 14 combinations) (see Table 1) used in the P30D, for each respective analysis.

#### Association between quantity of SP flavors used and smoking outcomes

Bivariate and multivariable linear regression was used to quantify the outcome of the association between quantity of fruit/other and mint flavored SP use and percent reduction in quantity of cigarettes smoked in the P30D. Bivariate and multivariable logistic regression was used to quantify the association between the quantity of fruit/other and mint flavored SP used and the likelihood of quitting vs. not quitting smoking. For bivariate analyses, the exposure variable was the total quantity of SP used in the P30D among participants using: (1) predominantly fruit/other flavored SP, (2) predominantly mint flavored SP, (3) exclusively fruit/other flavored SP, and (4) exclusively mint flavored SP. For the multivariable analyses, the exposure variables were the simultaneous use of: (1) the quantity of fruit/other flavored SP used and (2) the quantity of mint flavored SP used among all participants, regardless of predominant use.

## Results

### Study cohort

**Fig. 1:**
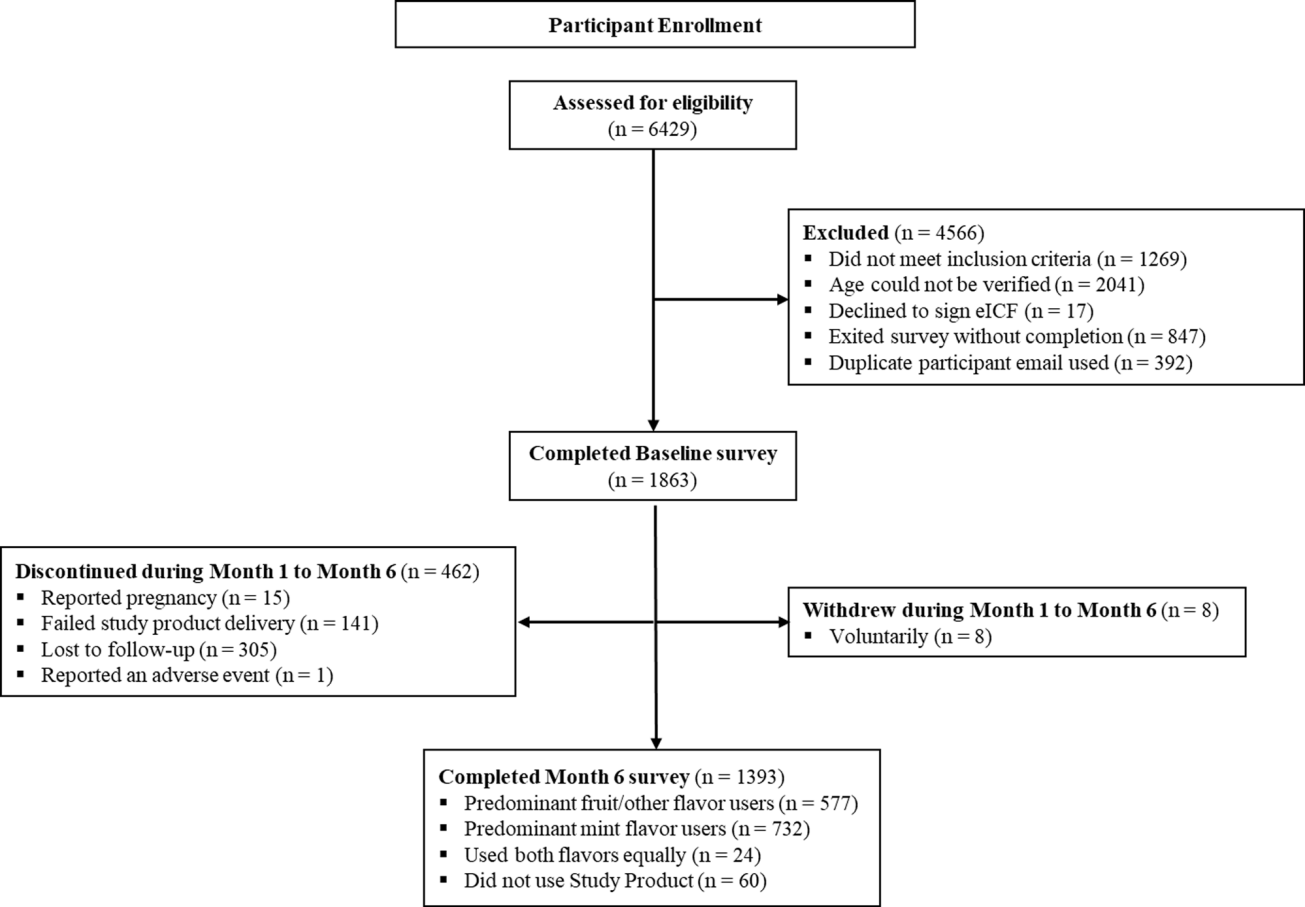
Participant Recruitment and Enrollment

Among the 1863 participants enrolled, 1393 (74.8%) completed the study (participated at Month 6). The 1393 participants were classified by the quantity of each SP flavor category they used at Month 6. This classification resulted in 577 (41.4%) predominant fruit/other users and 732 (52.5%) predominant mint users, 24 (1.7%) who used both equally, and 60 (4.3%) who reported they had not used any SP in the P30D. More details regarding recruitment and enrollment of the study cohort are found in Fig. 1.

All 1393 participants were included in descriptive analyses. There were 8 participants who responded *don’t know* when asked if they had smoked in the P30D, resulting in missing values for their smoking status at Month 6. These participants were excluded from bivariate/multivariable analyses, resulting in a final analytical cohort of 1385 participants. All predominant fruit/other and predominant mint users were complete cases with no missing data for smoking status.

### Participant demographics

Among the 1393 participants who completed the study, the proportion of males (50.3%) and females (49.4%) was about the same. Most participants were ages 35–54 years, White, and lived in the South (U.S. Census region). About two thirds of participants were currently employed, had an annual household income of less than $50,000, and had not attained a bachelor’s degree.

Results for demographic characteristics were generally the same when participants were classified based on the predominant SP flavor category, with a few notable differences. There was a greater proportion of predominant fruit/other users who were male (male: 54.1%; female: 45.6%). Otherwise, differences between the subgroups of flavor users for other demographic characteristics were small (Table 2).

**Table 2:** Demographic characteristics of participants at Month 6 (*n* = 1393)

	All participants (*n* = 1393, 100.0%)	Predom. fruit/other SP users (*n* = 577, 41.4%)	Predom. mint SP users (*n* = 732, 52.5%)	*p*-value (Fruit/other vs. mint users)^4^
**Demographic characteristic** ^**1**^				
Age, mean (SD)	46.4 (12.4)	45.8 (12.3)	46.8 (12.5)	0.148
Age Category, n (%)				
21–34 years	264 (19.0)	123 (21.3)	130 (17.8)	0.192
35–54 years	734 (52.7)	300 (52.0)	383 (52.3)	
55 + years	395 (28.4)	154 (26.7)	219 (29.9)	
Gender, n (%)				
Male	701 (50.3)	312 (54.1)	355 (48.5)	0.126
Female	688 (49.4)	263 (45.6)	375 (51.2)	
Non-binary/other	4 (0.3)	2 (0.3)	2 (0.3)	
Race, n (%)				
White or Caucasian	1164 (83.6)	492 (85.3)	602 (82.2)	0.085
Non-White^2^	222 (15.9)	80 (13.9)	128 (17.5)	
Black or African American	143 (10.3)	48 (8.3)	89 (12.2)	
Other or multiple races	79 (5.7)	32 (5.5)	39 (5.3)	
Unknown	7 (0.5)	5 (0.9)	2 (0.3)	
Ethnicity, n (%)				
Hispanic	109 (7.8)	42 (7.3)	61 (8.3)	0.487
Non-Hispanic	1283 (92.1)	534 (92.5)	671 (91.7)	
Geographic Region, n (%)^3^				
Midwest	352 (25.3)	153 (26.5)	171 (23.4)	0.622
Northeast	210 (15.1)	86 (14.9)	113 (15.4)	
South	638 (45.8)	259 (44.9)	346 (47.3)	
West	193 (13.9)	79 (13.7)	102 (13.9)	
Annual Household Income, n (%)				
Less than $50,000	813 (58.4)	337 (58.4)	431 (58.9)	0.983
$50,000 to $99,999	435 (31.2)	180 (31.2)	230 (31.4)	
$100,000 or more	127 (9.1)	49 (8.5)	65 (8.9)	
Employment Status, n (%)				
Currently employed	841 (60.4)	342 (59.3)	456 (62.3)	0.300
Not currently employed	550 (39.5)	233 (40.4)	276 (37.7)	
Level of Education, n (%)				
Less than a bachelor’s degree	889 (63.8)	381 (66.0)	456 (62.3)	0.162
Bachelor’s degree or higher	504 (36.2)	196 (34.0)	276 (37.7)	
Sexual Orientation, n (%)				
Heterosexual	1220 (87.6)	505 (87.5)	645 (88.1)	0.618
Not heterosexual	165 (11.8)	70 (12.1)	82 (11.2)	
Veteran Status, n (%)				
Currently on active duty	2 (0.1)	1 (0.2)	1 (0.1)	0.978
Formerly on active duty	121 (8.7)	50 (8.7)	65 (8.9)	
Not a veteran	1269 (91.1)	525 (91.0)	666 (91.0)	

^1^ Participants could respond *don’t know* or could decline to answer demographic questions on race, ethnicity, annual household income, employment status, level of education, sexual orientation, and veteran status. These data are not presented due to low incidence. Thus, percentages within a demographic characteristic may not sum to 100.0%

^2^ Non-White includes participants who reported they were Black, Native Hawaiian/Pacific Islander, Asian, another race, or reported multiple races

^3^ Derived from state of residence based on U.S. Census Bureau geographic region definitions

^4^ Continuous variables compared using Student’s unpaired t-tests; categorial variables were compared using chi-square tests

### Tobacco and nicotine product use at baseline

#### Cigarette smoking at Baseline

All participants (*n* = 1393) were current AS at Baseline per the inclusion criteria (91.7% smoked daily; 8.3% smoked nondaily). Participants smoked an average of 15.4 cigarettes per day (CPD). Baseline smoking frequency was generally similar among participants overall and among the subgroups of predominant fruit/other and mint SP users. At Baseline, more predominant fruit/other SP users reported they usually smoked nonmenthol cigarettes (63.1%) than did predominant mint SP users (50.3%) (*p* < 0.001) (Table 3).

#### Other tobacco product use at baseline

Among all participants (*n* = 1393), current use (daily or nondaily in P30D) of ENDS (44.6%) and non-cigarette smokeable tobacco products (36.4%) was more prevalent than all other categories of TNP assessed. Few participants were current users of ONP at Baseline (11.8%). Other noncigarette TNP use at Baseline was similar between predominant fruit/other and mint SP users (Supplemental Table A).

**Table 3:** Past-30-Day cigarette smoking at baseline and Month 6 (*n* = 1393)

	All participants^1^ (*n* = 1393, 100.0%)		Predom. fruit/other SP users (*n* = 577, 41.4%)		Predom. mint SP users (*n* = 732, 52.5%)		*p*-value (Fruit/other vs. mint users at month 6)^5^
	Baseline	Month 6	Baseline	Month 6	Baseline	Month 6	
**Cigarette smoking,** ** n (%)**							
Currently smoked	1393 (100.0)	1200 (86.2)	577 (100.0)	485 (84.1)	732 (100.0)	642 (87.7)	0.045
* Daily smokers*	*1277 (91.7)*	*753 (54.1)*	*529 (91.7)*	*295 (51.1)*	*672 (91.8)*	*397 (54.2)*	
* Nondaily smokers*	*116 (8.3)*	*447 (32.1)*	*48 (8.3)*	*190 (32.9)*	*60 (8.2)*	*245 (33.5)*	
Nonsmokers/quit smoking	----	185 (13.3)	----	89 (15.4)	----	85 (11.6)	
Don’t know	----	8 (0.6)	----	3 (0.5)	----	5 (0.7)	
**Type of cigarette smoked**,** n (%)**^**2**^							
Nonmenthol	780 (56.0)	----	364 (63.1)	----	368 (50.3)	----	< 0.001
Menthol	611 (43.9)	----	211 (36.6)	----	364 (49.7)	----	
Don’t know	2 (0.1)	----	2 (0.3)	----	0 (0.0)	----	
**Smoking frequency** ^**3**^							
Days smoked, mean (SD)	29.0 (3.6)	24.3 (8.9)	29.1 (3.5)	24.5 (8.6)	29.0 (3.6)	23.7 (9.3)	0.140
CPD, mean (SD)	15.4 (8.8)	10.3 (7.5)	16.2 (8.9)	10.2 (7.0)	14.8 (8.8)	10.0 (7.8)	0.656
**Smoking reduction**,** n (%)**^**3**^							
* ≥* 50% CPD reduction	----	524 (37.6)	----	224 (38.8)	----	288 (39.3)	0.847

^1^ All participants at Month 6 includes 41.4% (*n* = 577) predominant fruit/other flavor users, 52.5% (*n* = 732) predominant mint flavor users, 4.3% (*n* = 60) SP nonusers, and 1.7% (*n* = 24) participants who used equal amounts of fruit/other and mint flavors.

^2^ Type of cigarette smoked at Month 6 was based on responses provided at Baseline; thus, results are the same for both timepoints. Statistical comparison reflects difference in type of cigarette smoked at Baseline between predominant fruit/other and predominant mint users. Type of cigarette smoked was not assessed at Month 6.

^3^ Smoking frequency presented for those who were daily or nondaily smokers at Month 6; results at Month 6 do not include those who had quit smoking.

^4^ Current smoking was defined as those who had smoked daily or nondaily in the past 30 days at the respective timepoint.

^5^ Continuous variables compared using Student’s unpaired t-tests; categorial variables were compared using chi-square tests. Baseline versus Month 6: *p* < 0.001 for all comparisons.

### Study product use at Month 6

At Month 6, 95.7% of all 1393 participants reported use of SP in the P30D. A higher proportion of participants were predominant users of mint than fruit/other flavored SP (52.5% vs. 41.4%, respectively). There were 24 (1.7%) participants who reported using equal quantities of fruit/other and mint flavored SP and 60 (4.3%) participants who reported no SP used at Month 6. Participants on average used 7.7 pieces of any flavor per day. Predominant fruit/other users (6.4 pieces/day) and predominant mint users (6.3 pieces/day), on average, used similar numbers of pieces of their respective flavors per day. Predominant fruit/other users used a greater number of flavors in the P30D than predominant mint users (*p* < 0.003). There was no significant difference in the number of flavor-form combinations used between the groups (*p* = 0.118) (Table 4).

**Table 4:** Past-30-Day study product (SP) use at Month 6 (*n* = 1393)

	All participants^1^ (*n* = 1393, 100%)	Predom. fruit/other SP users (*n* = 577, 41.4%)	Predom. mint SP users (*n* = 732, 52.5%)	*p*-value (Fruit/other vs. mint users at month 6)^4^
**SP Use in P30D at Month 6**,** n (%)**	1333 (95.7%)	577 (100.0%)	732 (100.0%)	
**SP Use Frequency**,** mean (SD)**^**2**^				
Number of pieces of any flavor per day	7.7 (11.1)	8.0 (11.7)	8.0 (10.6)	> 0.999
* Pieces of fruit/other flavors per day*	*3.6 (6.8)*	*6.4 (9.2)*	*1.6 (3.1)*	< 0.001
* Pieces of mint flavors per day*	*4.1 (7.1)*	*1.6 (3.6)*	*6.3 (8.5)*	< 0.001
**Variety of SP Flavors Used**,** mean (SD)**				
Number of flavors varieties used	4.1 (2.5)	4.5 (2.5)	4.1 (2.4)	0.003
Number of flavor-form combinations used	5.2 (3.5)	5.3 (3.5)	5.6 (3.4)	0.118

^1^ All participants at Month 6 includes 41.4% (*n* = 577) predominant fruit/other flavor users, 52.5% (*n* = 732) predominant mint flavor users, 4.3% (*n* = 60) SP nonusers, and 1.7% (*n* = 24) participants who used equal amounts of fruit/other and mint flavors.

^2^ SP use frequency presented for those who had used any SP in the past 30 days at Month 6.

^3^ A piece of SP is one pouch, one piece of gum, or one lozenge.

^4^ Continuous variables compared using Student’s unpaired t-tests.

### Tobacco and nicotine product use at month 6

#### Cigarette smoking at month 6

Overall, 13.3% of participants had quit smoking at Month 6. A higher proportion of predominant fruit/other SP users had quit smoking (15.4%) than did predominant mint users (11.6%) (*p* = 0.045). Those who had not quit smoking markedly reduced their cigarette smoking frequency at Month 6. About one third of participants among each flavor user group were nondaily smokers. A similar proportion of predominant fruit/other users (38.8%) and predominant mint users (39.3%) reduced the average quantity of cigarettes they smoked per day by *≥* 50% (*p* = 0.847) (Table 3).

#### Other tobacco and nicotine product use at month 6

There were no significant differences in other TNP (ENDS, smokeless tobacco, oral nicotine products, NRT, or non-cigarette smokeable tobacco products) use between predominant fruit/other and predominant mint users at Month 6 (*p* > 0.05 for all comparisons). Compared to Baseline, there was a significant decrease in use of ENDS and non-cigarette smokeable tobacco products at Month 6 among both user groups (*p* < 0.01 for all comparisons) (Supplemental Table A).

### Flavors of study products used and associated smoking reduction or quitting

#### Cigarette smoking reduction by Flavor

Use of SP at Month 6 was significantly associated with smoking reduction from Baseline, with an average percent reduction in CPD of 49.7% overall (*p* < 0.001). In addition, use of a greater number of SP flavor varieties was significantly associated with smoking reduction (2.2% greater reduction per additional flavor [95% CI: 1.4-3.0%]; *p* < 0.001). The same was true for greater numbers of flavor-form combinations used (1.4% greater reduction per additional flavor-form [95% CI: 0.8-2.0%]; *p* < 0.001) (Table 5).

An increased quantity of fruit/flavored SP used was independently associated with smoking reduction in a multivariable model (8.6% greater reduction per 10 fruit/other pieces/day [95% CI: 5.3–11.9%]; *p* < 0.001) adjusting for quantity of mint use. Similarly, an increased quantity of mint flavored SP used was independently associated with smoking reduction (7.5% greater reduction per 10 mint pieces/day [95% CI: 4.4–10.6%]; *p* < 0.001) adjusting for quantity of fruit/other use (Table 5).

An increased quantity of any flavor of SP used was significantly associated with smoking reduction (8.0% greater reduction per 10 pieces/day [95% CI: 6.0–10.0%]; *p* < 0.001). Results were similar for predominant and exclusive users of each flavor category, though the magnitude of the association was higher for exclusive users than for predominant users (*p* < 0.001 for each flavor user subgroup) (Table 5).

**Table 5:** Percent reduction in cigarette smoking by flavors of study product (SP) used at Month 6 (*n* = 1385)

	Percent reduction in cigarette smoking at Month 6				
	Average reduction, %	Incremental reduction, %	95% CI		*P*-value
			Lower, %	Upper, %	
**Participants With Smoking Status,** *** n*** ** = 1385**	49.7				
Number of SP flavor varieties used					
+ Per additional flavor		2.2	1.4	3.0	< 0.001
Number of SP flavor-form combinations used					
+ Per additional combination		1.4	0.8	2.0	< 0.001
Quantity of SP used per day					
+ Per 10 pieces of any flavor		8.0	6.0	10.0	< 0.001
Quantity of SP used per day by flavor					
+ Per 10 pieces of fruit/other flavors		8.6	5.3	11.9	< 0.001
+ Per 10 pieces of mint flavors		7.5	4.4	10.6	< 0.001
**Classified by Predominant Flavor Use**					
**Predominant Fruit/Other SP Users,** *** n*** ** = 577**	50.0				
Quantity of SP used per day					
+ Per 10 pieces of any flavor		6.8	3.7	10.0	< 0.001
**Predominant Mint SP Users,** *** n*** ** = 732**	48.4				
Quantity of SP used per day					
+ Per 10 pieces of any flavor		7.8	5.0	10.6	< 0.001
**Classified by Exclusive Flavor Use**					
**Exclusive Fruit/Other SP Users,** *** n*** ** = 191**	39.6				
Quantity of SP used per day					
+ Per 10 pieces of fruit/other flavors		12.7	5.7	19.6	< 0.001
**Exclusive Mint SP Users,** *** n*** ** = 174**	38.2				
Quantity of SP used per day					
+ Per 10 pieces of mint flavors		16.4	9.0	23.9	< 0.001

#### Quitting cigarette smoking by flavor

At Month 6, 13.3% of participants had quit smoking (*p* < 0.001). Using more SP flavor varieties or form-flavor combinations was not associated with increased odds of quitting smoking (Table 6).

An increased quantity of fruit/flavored SP used was independently associated with increased odds of quitting smoking in a multivariable model (OR = 1.29 [95% CI: 1.04–1.59] per 10 fruit/other pieces used/day; *p* = 0.017) adjusting for quantity of mint use. However, an increased quantity of mint flavored SP used was not independently associated with increased odds of quitting smoking (OR = 1.04 [95% CI: 0.82–1.28] per 10 mint pieces used/day; *p* = 0.75), adjusting for quantity of fruit/other use (Table 6).

Use of a greater quantity of SP was associated with significantly increased odds of quitting smoking among all participants (OR = 1.16 [95% CI: 1.01–1.32] per 10 pieces used/day; *p* < 0.001). This significant association was also found among predominant fruit/other users (OR = 1.24 [95% CI: 1.02–1.49] per 10 pieces used/day; *p* = 0.029) but was nonsignificant among predominant mint users, exclusive fruit/other users, and exclusive mint users (Table 6).

**Table 6:** Odds of quitting cigarette smoking by flavors of Study products (SP) used at Month 6

	Odds of quitting cigarette smoking at Month 6				
	Quit smoking, n (%)	Odds ratio	95% CI of odds ratio		*p*-value
			Lower	Upper	
**Participants Reporting Smoking Status,** *** n*** ** = 1385**	185 (13.3)				
Number of SP flavor varieties used					
+ Per additional flavor		0.97	0.91	1.04	0.41
Number of SP form-flavor combinations used					
+ Per additional combination		0.96	0.92	1.01	0.11
Quantity of SP used per day					
+ Per 10 pieces of any flavor		1.16	1.01	1.32	< 0.001
Quantity of SP used per day by flavor					
+ Per 10 pieces of fruit/other flavors		1.29	1.04	1.59	0.017
+ Per 10 pieces of mint flavors		1.04	0.82	1.28	0.75
**Classified by Predominant Flavor Use**					
**Predominant Fruit/Other SP Users,** *** n*** ** = 577**	89 (15.4)				
Quantity of SP used per day					
+ Per 10 pieces of any flavor		1.24	1.02	1.49	0.029
**Predominant Mint SP Users,** *** n*** ** = 732**	85 (11.6)				
Quantity of SP used per day					
+ Per 10 pieces of any flavor		1.06	0.84	1.29	0.59
**Classified by Exclusive Flavor Use**					
**Exclusive Fruit/Other SP Users,** * n* ** = 191**	39 (20.4)				
Quantity of SP used per day					
+ Per 10 pieces of fruit/other flavors		1.22	0.79	1.81	0.33
**Exclusive Mint SP Users,** *** n*** ** = 174**	16 (9.2)				
Quantity of SP used per day					
+ Per 10 pieces of mint flavors		0.96	0.41	1.81	0.92

## Discussion

The purpose of the present analysis was to understand the degree of association between the use of flavored ONP and smoking reduction and quitting over six months and whether there were differences by fruit/other or mint flavor use (which represent nontraditional and traditional flavors, respectively, for the greater oral/smokeless tobacco category). In summary, results showed that using a greater number of SP flavor varieties was significantly associated with greater smoking reduction but not odds of quitting. Predominant fruit/other flavored SP users had higher levels of cigarette reduction, and a higher proportion had quit smoking than predominant mint flavored SP users. Increased use of fruit/other flavored SP was independently associated with greater smoking reduction and odds of quitting, whereas increased use of mint flavored SP was independently associated with smoking reduction but not with odds of quitting. Further, results suggest the use of nontraditional oral/smokeless tobacco flavors, such as fruit/other flavors, have an increased effect on cigarette reduction and quitting than use of traditional flavors, such as mint flavors.

These findings are consistent with prior literature, where use of novel, flavored TNP has been shown to facilitate smoking reduction and quitting. AS who fully switched to ENDS have been shown to use a greater variety of flavors than those who remained dual users [17, 23, 25]. Results of a systematic literature review on use of flavored ENDS among current and former AS suggests that availability of a variety of nontobacco and nonmenthol flavors facilitates complete switching from cigarettes [40]. Becker et al., found that using more ONP (nicotine pouches) flavor varieties over 6-weeks of *ad libitum* use was associated with a decrease in cigarette consumption from baseline. The authors did not report cigarette reduction across flavors or flavor categories [36]. This result was similar to our findings, as the number of SP flavor varieties used at Month 6 was significantly associated with smoking reduction.

There is some debate on the differing impact of fruit/other flavor use relative to mint flavors on switching from cigarette use. In these analyses, fruit/other flavor use, but not mint flavor use, was associated with significantly increased odds of quitting smoking. The differential effect on switching behavior from using fruit/other versus mint flavors is also supported elsewhere. For example, adult smokers who used flavored TNP were more likely to quit smoking compared to those who used tobacco, unflavored, or menthol flavors [17, 26, 41]. Results from a study on using flavored, non-nicotine gum to curb nicotine withdrawal while making a quit attempt suggested using non-mint flavors reduced withdrawal symptoms but found no effect for use of mint-flavored gum [42].

There are strengths and limitations to the present study and analyses that should be considered. The duration of the AUP allowed participants to adopt use of the SP in their own environment and to sample the variety of available flavor-form combinations, if desired. Determining reduction and quitting at Month 6 also established evidence of longer-term changes in smoking behaviors, relative to evidence from existing literature on ONP use. While changes in smoking behaviors are multifactorial (e.g., psychosocial, economic factors), the levels of cigarette reduction and quitting observed among participants in this observational, actual use study suggest that use of commercially available, flavored ONP may be associated with changes in smoking behaviors. The SP were provided at no cost to participants which may have contributed to higher rates of continued participation and SP use [43], but the effect on smoking reduction and quitting in this study is unknown. The study relied on self-reported use behaviors that can be subject to recall bias, and there was no biochemical confirmation of cigarette abstinence given the non-site-based approach. Potential effects of these limitations on the results are not expected to have differed between the SP flavor categories.

## Conclusion

Increased use of either fruit/other or mint flavored Study Products at Month 6 was associated with significantly increased smoking reduction whereas only increased use of fruit/other flavors was associated with greater odds of quitting smoking among participants in the study.

## Electronic supplementary material

Below is the link to the electronic supplementary material.

Supplementary Material 1

## Data Availability

The dataset supporting the conclusions of this article are available from Oracle Life Sciences, Oracle Corporation, but restrictions apply to the availability of these data and so they are not publicly available. Data are, however, available from the corresponding author upon reasonable request and per the express approval and permission of Oracle Life Sciences.

## Abbreviations

AS: Adults who smoke cigarettes (adult smokers)
AUP: Actual use period
CI: Confidence interval
CPD: Cigarettes per day
ENDS: Electronic nicotine delivery systems
FDA: United States Food and Drug Administration
NRT: Nicotine replacement therapies
ONP: Oral nicotine products
OR: Odds ratio
P30D: Past 30 days
PATH: Population Assessment of Tobacco and Health Study
SP: Study Products
SD: Standard deviation
TNP: Tobacco and nicotine products
U. S.: United States
