# Supplementary material for: Effect of fruit and mint flavored Rogue® oral nicotine product use on smoking reduction and quitting in a 6-Month prospective cohort of adults who smoke cigarettes
Source: BMC Public Health. 2024 Nov 22;24:3249. doi: 10.1186/s12889-024-20463-3 (PMC11583794; doi:10.1186/s12889-024-20463-3)
Supplement: Supplementary file 1 — Supplementary Material 1 [file 12889_2024_20463_MOESM1_ESM.docx]

**Supplemental Table A**

|  | **All Participants** (n=1393, 100.0%) | | **Predom. Fruit/Other Users**  (n=577, 41.4%) | | **Predom. Mint Users**  (n=732, 52.5%) | | **p-value**  (Fruit/Other vs. Mint Users at Month 6)^1,2^ |
| --- | --- | --- | --- | --- | --- | --- | --- |
|  | Baseline | Month 6 | Baseline | Month 6 | Baseline | Month 6 |  |
| **Electronic Nicotine Delivery Systems, n (%)** | | | | | | |  |
| Daily | 153 (11.0) | 114 (8.2) | 60 (10.4) | 51 (8.8) | 84 (11.5) | 61 (8.3) | 0.447 |
| Nondaily | 468 (33.6) | 312 (22.4) | 205 (35.5) | 139 (24.1) | 249 (34.0) | 156 (21.3) |  |
| Not at all | 757 (54.3) | 926 (66.5) | 308 (53.4) | 371 (64.3) | 390 (53.3) | 492 (67.2) |  |
| Don't know | 15 (1.1) | 41 (2.9) | 4 (0.7) | 16 (2.8) | 9 (1.2) | 23 (3.1) |  |
| **Smokeless Tobacco Products, n (%)** | | | | | | |  |
| Daily | 31 (2.2) | 63 (4.5) | 15 (2.6) | 35 (6.1) | 16 (2.2) | 28 (3.8) | 0.097 |
| Nondaily | 130 (9.3) | 216 (15.5) | 58 (10.1) | 100 (17.3) | 69 (9.4) | 110 (15.0) |  |
| Not at all | 1214 (87.2) | 1054 (75.7) | 499 (86.5) | 422 (73.1) | 635 (86.7) | 556 (76.0) |  |
| Don't know | 18 (1.3) | 60 (4.3) | 5 (0.9) | 20 (3.5) | 12 (1.6) | 38 (5.2) |  |
| **Oral Nicotine Products (Non-NRT), n (%)** | | | | | | |  |
| Daily | 14 (1.0) | 449 (32.2) | 5 (0.9) | 212 (36.7) | 9 (1.2) | 230 (31.4) | 0.081 |
| Nondaily | 151 (10.8) | 674 (48.4) | 60 (10.4) | 283 (49.0) | 87 (11.9) | 374 (51.1) |  |
| Not at all | 1204 (86.4) | 253 (18.2) | 503 (87.2) | 76 (13.2) | 623 (85.1) | 119 (16.3) |  |
| Don't know | 24 (1.7) | 17 (1.2) | 9 (1.6) | 6 (1.0) | 13 (1.8) | 9 (1.2) |  |
| **Nicotine Replacement Therapy, n (%)** | | | | | | |  |
| Daily | 10 (0.7) | 93 (6.7) | 6 (1.0) | 36 (6.2) | 4 (0.5) | 53 (7.2) | 0.550 |
| Nondaily | 128 (9.2) | 253 (18.2) | 44 (7.6) | 103 (17.9) | 81 (11.1) | 143 (19.5) |  |
| Not at all | 1229 (88.2) | 971 (69.7) | 519 (89.9) | 404 (70.0) | 631 (86.2) | 496 (67.8) |  |
| Don't know | 26 (1.9) | 76 (5.5) | 8 (1.4) | 34 (5.9) | 16 (2.2) | 40 (5.5) |  |
| **Non-Cigarette Smokeable Tobacco Products, n (%)** | | | | | | |  |
| Daily | 132 (9.5) | 108 (7.8) | 53 (9.2) | 38 (6.6) | 71 (9.7) | 62 (8.5) | 0.159 |
| Nondaily | 375 (26.9) | 238 (17.1) | 158 (27.4) | 110 (19.1) | 202 (27.6) | 115 (15.7) |  |
| Not at all | 869 (62.4) | 979 (70.3) | 363 (62.9) | 401 (69.5) | 450 (61.5) | 518 (70.8) |  |
| Don't know | 17 (1.2) | 68 (4.9) | 3 (0.5) | 28 (4.9) | 9 (1.2) | 37 (5.1) |  |
| ^1^ Statistical comparison between predominant fruit/other and predominant mint users at Month 6. Categorical variables were compared using chi-square tests.  ^2^ All comparisons between difference between Baseline and Month 6 p<0.001 among both user groups. | | | | | | | |
